# Bleb Compressive Sutures in Descemet Stripping Automated Endothelial Keratoplasty for Eyes with Filtering Blebs Following Trabeculectomy

**DOI:** 10.3390/jcm15093439

**Published:** 2026-04-30

**Authors:** Noriko Toyokawa, Kaoru Araki-Sasaki, Hideya Kimura, Shinichiro Kuroda

**Affiliations:** 1 Nagata Eye Clinic, 1147 Kitayamada Horai, Nara City 631-0844, Nara, Japan; sasakis@sa2.so-net.ne.jp (K.A.-S.);; 2 Department of Ophthalmology, Kansai Medical University, 2-5-1 Shinmachi, Hirakata City 573-1010, Osaka, Japan

**Keywords:** DSAEK, glaucoma filtration surgery, air tamponade, graft dislocation, bleb leak, bleb damage

## Abstract

**Background/Objectives**: A disadvantage of Descemet stripping automated endothelial keratoplasty (DSAEK) in eyes with prior glaucoma filtration surgery is the difficulty in maintaining air tamponade during the procedure. Herein, we report the use of bleb compressive sutures in managing air tamponade in the anterior chamber during DSAEK in eyes with blebs following trabeculectomy. **Methods**: This retrospective case series included 34 eyes of 33 patients that developed bullous keratopathy following trabeculectomy. Bleb compression suturing was performed using a 10-0 nylon suture in eyes with an intraocular pressure (IOP) < 10 mmHg or a fragile ischemic bleb. Postoperative IOP, air ingress into the bleb, rebubbling, bleb leakage, and bleb damage were evaluated. **Results**: Of the 34 eyes, 13 underwent bleb compression suturing before DSAEK (suture group), whereas 21 eyes did not (non-suture group). The mean preoperative IOP was 7.5 ± 2.5 mmHg and 11.2 ± 4.2 mmHg in the suture and the non-suture groups, respectively. The IOP was measured 2 h postoperatively in 14 eyes, increasing by 18 ± 9.3 and 11.7 ± 3.1 mmHg in the suture and non-suture groups, respectively, compared to the preoperative IOP, with no significant differences. At 2 h postoperatively, two eyes in the suture group and one eye in the non-suture group exhibited an IOP spike (≥30 mmHg). One eye in the non-suture group required rebubbling owing to air ingress into the bleb. The mean IOP was 7.1 ± 3.2 and 9.4 ± 4.6 mmHg in the suture and non-suture groups, respectively, 1–2 weeks postoperatively. Preoperative and postoperative IOPs did not significantly differ in either group, and no suture-related complications were observed. **Conclusions**: For eyes with blebs, bleb compression suturing in DSAEK provides effective air tamponade during graft adhesion.

## 1. Introduction

Bullous keratopathy (BK) is a serious surgical complication that occurs following glaucoma surgery, including following trabeculectomy and glaucoma drainage device implantation [1]. Descemet stripping endothelial keratoplasty (DSAEK) has emerged as a viable technique for treating BK following glaucoma filtering surgery [2,3,4]. However, prior glaucoma filtering surgery represents a significant risk factor for graft failure [4,5,6,7,8,9]. Multiple factors contribute to graft failures, such as altered anatomy of the anterior chamber, difficulty in maintaining air tamponade during the operation and in the immediate postoperative period [6,9], chronic subclinical inflammation due to disruption in the blood–aqueous barrier, and persistent direct communications between the anterior chambers [10]. Goshe et al. also reported that graft dislocation is closely linked to the presence of postoperative hypotony [11]. Notably, maintaining a sufficiently high intraocular pressure (IOP) during the early postoperative period is crucial for ensuring the proper adhesion of the implanted graft to the stroma [11,12,13,14]. This is particularly challenging in eyes with previous trabeculectomy, as they often exhibit a low IOP or hypotony due to overfiltration and/or a compromised ciliary body function [15].

In the present report, we demonstrate that the use of bleb compressive bridging sutures can lead to an adequate IOP during DSAEK in patients with blebs following trabeculectomy. In some cases, performing anterior chamber air filling in eyes with filtering blebs was challenging; therefore, we instituted a protocol of placing bleb compressive bridging sutures in these eyes to prevent air from escaping from the anterior chamber into the bleb.

## 2. Materials and Methods

The study protocol was approved by the Nagata Eye Clinic Institutional Review Board (#2025-003) and adhered to the tenets of the Declaration of Helsinki. Written informed consent was obtained from all patients.

A retrospective chart review was conducted for all consecutive patients who underwent DSAEK for BK in glaucomatous eyes with previous trabeculectomy between February 2014 and May 2025 at the Nagata Eye Clinic. All eyes with prior trabeculectomies were identified and designated for this analysis. Patients with less than six months of follow-up after DSAEK were excluded from the study.

### 2.1. Surgical Procedures and Postoperative Management

All DSEAK procedures were performed by two experienced surgeons (KAS and SK), and bleb compressive suturing was performed by two glaucoma specialists (NT and SK).

The general surgical technique was based on the currently accepted standard practice. An anterior chamber maintainer was inserted inferiorly and temporally into the anterior chamber. A temporal 5.0 mm clear corneal incision was made so that incision site did not interfere with the bleb. A peripheral iridectomy was performed in the inferior part of the iris to prevent postoperative pupillary block. The graft was inserted using a pull-through technique with the Busin glide (Moria, Doylestown, PA, USA). First, pupil size air was injected into the anterior chamber and the graft was centered using an Ambler LASIK/DSAEK roller (Ambler Surgical, Exton, PA, USA). Then, a sufficient amount of additional air was injected into the anterior chamber. The residual interface fluid was removed via drainage through the corneal venting incisions. Intraoperative manipulation of goniosynechialysis was minimized to prevent excessive postoperative inflammation.

Compressive bridging mattress sutures were placed on the bleb in eyes with an IOP of less than 10 mmHg or in the presence of a fragile cystic ischemic bleb prior to starting DSAEK. For bleb compressive sutures, a 10-0 nylon suture was passed through the conjunctiva and episclera to compress the bleb surface toward the underlying sclera. One or two mattress sutures were placed.

**Figure 1:**
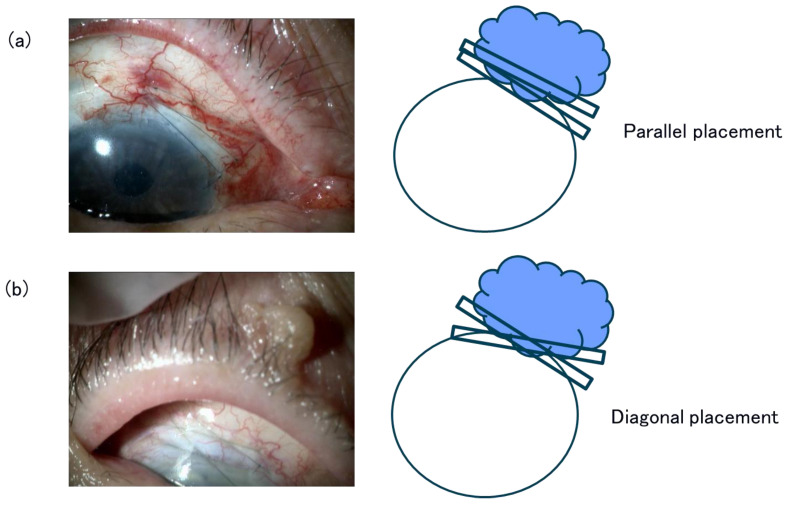
Slit lamp photographs and schematic images of compressive 10-0 nylon sutures for a non-ischemic low bleb. Compressive bridging sutures were placed to compress the bleb adjacent to the trabeculectomy filter (sclerostomy area). (**a**) Two compressive mattress sutures are placed in parallel. (**b**) Two compressive mattress sutures are placed diagonally.

**Figure 2:**
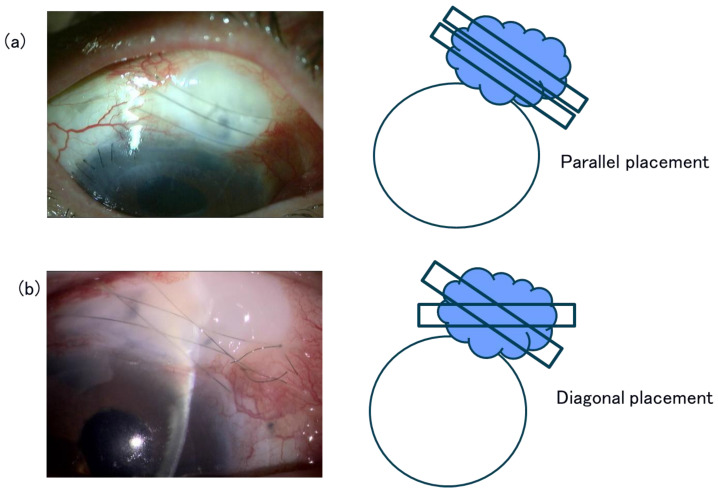
Slit lamp photographs and schematic images of compressive10-0 nylon sutures for a fragile ischemic high bleb. Compressive bridging sutures were placed to compress the entire ischemic thin bleb. Special care is required to avoid injury to the fragile ischemic blebs during suturing. (**a**) Two compressive mattress sutures are placed parallel to the horizontal boundary of the bleb. (**b**) Two compressive mattress sutures were placed diagonally over the horizontal boundary of the bleb.

The design, location, and number of compressive sutures of 10-0 nylon were determined according to the bleb morphology (Figure 1 and Figure 2). We evaluated the blebs based on their height, extent and vascularity using the Indiana Bleb Appearance Grading Scale [16]. We classified the blebs into two categories: non-ischemic low blebs with a low height (lower than one corneal thickness), a horizontal extent of less than 1–2 clock hours and mild vascularity, and ischemic high blebs, which were considered as fragile, with a moderate height (greater than 1.5 times the thickness of the cornea) and a horizontal extent of more than 2 clock hours and which were ischemic.

For non-ischemic low blebs, one or two sutures were placed adjacent to the trabeculectomy filter (sclerostomy area) to compress the bleb locally (Figure 1). For ischemic high blebs (fragile blebs), two compressive sutures were placed to compress the entire fragile ischemic bleb (Figure 2).

IOP was measured 2 h postoperatively to confirm that pupillary block had not occurred.

Leakage was assessed using a fluorescein strip viewed through cobalt-blue filter illumination in the Seidel test. The topical steroid dose was gradually tapered down from 4 times a day to once daily over a period of 4–6 months. Topical glaucoma medication was resumed postoperatively as needed. No air removal was attempted in any case.

### 2.2. Data Collection and Analysis

Postoperative demographics, ocular history, previous surgery, glaucoma history, intraoperative and postoperative outcomes, and surgical complications, including air ingress into the bleb, air rebubbling, bleb leak, bleb rupture, and bleb-related infection, were retrospectively reviewed from the patient charts. A paired *t*-test was used for preoperative and postoperative comparisons of IOP. Differences between the groups were assessed using the Mann–Whitney U test or Fisher’s exact test, as appropriate. IOP was measured using a rebound tonometer (iCare; Tiolat Oy, Espoo, Finland) or a Goldmann applanation tonometer (Haag-Streit, Köniz, Switzerland). Data were analyzed using StatMate Ver5 software (AtmsCorp, Tokyo, Japan). Statistical significance was set at *p* < 0.05.

## 3. Results

The study group comprised 34 eyes of 33 patients. These patients included 28 men and 5 women. The mean age at the time of DSAEK was 72.5 ± 9.2 years. All 34 eyes were pseudophakic, and bleb compressive bridging sutures were placed in 13 eyes (suture group), whereas the remaining 21 eyes did not receive sutures (non-suture group). The mean follow-up period after DSAEK was 32 ± 28 months. The IOP was controlled with or without glaucoma medication in all eyes prior to DSAEK.

### 3.1. Postoperative IOP in the Acute (2 h After DSAEK) and Chronic Phases (A Few Weeks After DSAEK)

Postoperative IOP was measured 2 h after DSAEK in 14 eyes, with 7 eyes each in each group. Due to the unavailability of the IOP measurement tool at the bedside, postoperative IOP at 2 h was not measured in all eyes. The mean preoperative IOP was 8.4 ± 3.0 mmHg in the suture group and 11.9 ± 3.6 mmHg in the non-suture group, and the mean postoperative IOP 2 h after DSAEK was 26.1 ± 8.5 mmHg (11–36 mmHg) and 25.0 ± 3.5 mmHg (21–31 mmHg) in these groups, respectively. The mean IOP increase from the preoperative phase to 2 h postoperatively was 18 ± 9.3 mmHg in the suture group and 11.7 ± 3.1 mmHg in the non-suture group. Although the mean increase in IOP tended to be greater in the suture group, the difference between the groups was not statistically significant (*p* = 0.26).

An IOP spike of ≥30 mmHg occurred in two eyes in the suture group and one eye in the non-suture group, with no significant difference between the groups. No postoperative pupillary block was observed, and the IOP decreased to below 20 mmHg by postoperative day 1 in all cases. Furthermore, the mean postoperative IOP at 1–2 weeks following DSAEK was 7.1 ± 3.2 mmHg in the suture group and 9.4 ± 4.6 mmHg in the non-suture group, indicating no statistically significant difference between preoperative and postoperative IOP. Notably, none of the eyes exhibited IOP instability during the postoperative follow-up, and no patient required new glaucoma medications for IOP control.

### 3.2. Air Ingress into the Filtration Bleb

Air ingress into the filtration bleb was observed only in one eye in the non-suture group.

### 3.3. Air Rebubbling and Graft Attachment Rate

The graft attachment rate was 84.6% (11/13 eyes) in the suture group and 90.5% (19/21 eyes) in the non-suture group. Rebubbling due to graft detachment was required in four eyes (two eyes each in each group), resulting in successful graft reattachment. Among these four eyes, one exhibited air ingress into the bleb during DSAEK. The incidence of rebubbling for graft detachment did not significantly differ between the two groups (*p* = 0.5).

### 3.4. Intraoperative Complications

No significant intraoperative complications, such as posterior dislocation of the donor DSAEK button, intraocular hemorrhage, suprachoroidal hemorrhage, bleb leak, bleb rupture, or bleb-related infection, occurred.

### 3.5. Removal of Compressive Sutures

**Figure 3:**
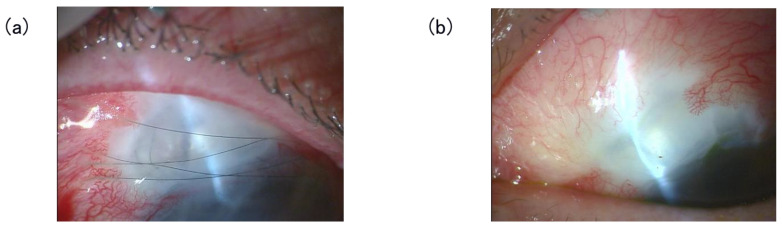
Before and after compressive suture removal. (**a**) Slit lamp photograph showing compressive 10-0 nylon bleb sutures. (**b**) Slit lamp photograph showing a functioning bleb after compressive suture removal.

Suture removal was performed in cases where suture loosening or a tendency to embed into the bleb was observed (6/13 eyes). In both suture removal and retention cases, no suture-related complications, including IOP instability, aqueous humor leakage from the bleb, bleb damage, or bleb-related infections, were observed (Figure 3).

## 4. Discussion

Prior glaucoma filtering surgery is associated with an increased risk of postoperative graft detachment after DSAEK [6,11,12]. This may be attributed to the presence of a filtering bleb which reduces the IOP. A low IOP, which allows fluid to enter the graft interface, leads to graft detachment [12,13]. We encountered similar situations in which it was difficult to achieve firm anterior chamber air filling in eyes with filtering blebs. Therefore, in the present study, we report the use of bleb compressive bridging sutures to maintain an adequate IOP during DSAEK in patients with prior trabeculectomy.

Maintaining an adequately high IOP during and immediately after surgery is essential for graft attachment [12,13,14]. In eyes with blebs, aqueous humor and air in the anterior chamber may readily escape into the filtering bleb. Therefore, several surgical approaches to maintain IOP during the early postoperative period in patients undergoing DSAEK with prior filtering surgery have been reported. For example, Banitt et al. achieved this by injecting air into the anterior chamber until the subconjunctival space was filled [12]. Moreover, Oyakawa et al. injected ophthalmic viscoelastic devices into the filtering bleb to achieve a sufficiently high IOP following DSAEK [13]. However, as blebs often become thin and fragile following antimetabolite use, any surgical procedure may increase the risk of bleb damage. Notably, bleb leakage and rupture are recognized as surgical complications in DSAEK [11,14]. Large-volume air injection increases the risk of barotrauma of thin, cystic blebs, potentially leading to their dysfunction [11]. Despite additional bleb suturing, bleb leakage and associated hypotony remain common complications following trabeculectomy [15]. Therefore, compression suturing should be performed with strategic and careful consideration of the suture placement site. In the present study, our approach to determine the suture site was as follows: in non-ischemic low blebs, compressive sutures were placed adjacent to the trabeculectomy filter (sclerostomy area) to compress the bleb, whereas in eyes with ischemic, fragile high blebs, compressive sutures were placed to compress the entire bleb. Overall, given the fragility of these blebs, it is crucial to avoid bleb injuries due to suture needles.

Bleb compression suturing has been applied in cases of persistent hypotony or bleb leakage following glaucoma filtration surgery [17,18]. This technique can help maintain complete air filling of the anterior chamber, resulting in a temporary increase in IOP and protection of the filtration bleb from barotrauma. In the present study, the IOP was significantly higher 2 h postoperatively in the suture group compared to the preoperative value (26.1 ± 8.5 mmHg; range 11–36 mmHg; *p* = 0.004). These findings suggest that bleb compression suturing provides effective air tamponade during the critical period of graft adhesion. Notably, this technique’s advantages are that it does not require any specialized surgical instruments and that it has broad applicability to eyes with a history of filtration surgery. However, extreme caution must be exercised when placing compression sutures in eyes with thin and/or fragile ischemic blebs to avoid bleb damage.

This study has certain limitations, including its retrospective design, small sample size, and lack of standardization for compression suturing and suture removal.

Based on our clinical experience regarding the indications for compression suturing, we adopted a threshold of 10 mmHg in this study; however, this may constitute a selection bias.

Furthermore, compression sutures were not performed in some cases despite the patient having an IOP below 10 mmHg because we judged the risk of air ingressing into the bleb to be low, as the blebs in these cases were relatively flat, thick-walled, and non-ischemic. Although we need to examine more cases in future to establish an IOP criterion, the intention of reporting this study is to propose this method as a means of avoiding poor graft adhesion due to a low IOP. Regarding the general key outcomes of DSAEK, long-term graft survival was not evaluated because the mechanisms of graft failure in eyes with blebs are multifactorial and the follow-up period for 10 (29.4%) of the studied eyes was shorter than 24 months. In addition, we did not evaluate visual acuity due to poor vision resulting from advanced glaucoma in most cases. The aim was to report the early results of compressive sutures to prevent air ingress into blebs.

In conclusion, this report demonstrates the effectiveness of bleb compressive bridging sutures in achieving an adequate IOP during DSAEK in eyes with a history of trabeculectomy. This technique appears to be safe and effective for eyes with blebs undergoing DSAEK, warranting further prospective studies.

## Data Availability

Data is contained within the article.

## Abbreviations

The following abbreviations are used in this manuscript:

DSAEK	Descemet stripping automated endothelial keratoplasty
BK	Bullous keratopathy
IOP	Intraocular pressure
